# Beneficial effects of a decreased meal frequency on nutrient utilization, secretion of luteinizing hormones and ovarian follicular development in gilts

**DOI:** 10.1186/s40104-021-00564-4

**Published:** 2021-04-06

**Authors:** Lun Hua, Lianpeng Zhao, Zhengyu Mao, Wentao Li, Jing Li, Xuemei Jiang, Lianqiang Che, Shengyu Xu, Yan Lin, Zhengfeng Fang, Bin Feng, De Wu, Yong Zhuo

**Affiliations:** 1 Animal Nutrition Institute, Sichuan Agricultural University, Chengdu, 611130 People’s Republic of China; 2 Key Laboratory for Animal Disease-Resistant Nutrition of the Ministry of Education of China, Sichuan Agricultural University, Chengdu, 611130 People’s Republic of China; 3 Key Laboratory of Animal Disease-Resistant Nutrition of Sichuan Province, Sichuan Agricultural University, 211 Huimin Road, Wenjiang District, Chengdu, 611130 People’s Republic of China

**Keywords:** Gilts, Luteinizing hormone, Meal frequency, Nutrient utilization, Ovarian follicular development

## Abstract

**Background:**

Replacement gilts are typically fed *ad libitum*, whereas emerging evidence from human and rodent studies has revealed that time-restricted access to food has health benefits. The objective of this study was to investigate the effect of meal frequency on the metabolic status and ovarian follicular development in gilts.

**Methods:**

A total of 36 gilts (Landrace × Yorkshire) with an age of 150±3 d and a body weight of 77.6±3.8 kg were randomly allocated into one of three groups (*n* = 12 in each group), and based on the group allocation, the gilts were fed at a frequency of one meal (T1), two meals (T2), or six meals per day (T6) for 14 consecutive weeks. The effects of the meal frequency on growth preference, nutrient utilization, short-chain fatty acid production by gut microbial, the post-meal dynamics in the metabolic status, reproductive hormone secretions, and ovarian follicular development in the gilts were measured.

**Results:**

The gilts in the T1 group presented a higher average daily gain (+ 48 g/d, *P* < 0.05) and a higher body weight (+ 4.9 kg, *P* < 0.05) than those in the T6 group. The meal frequency had no effect on the apparent digestibility of dry matter, crude protein, ether extract, ash, and gross energy, with the exception that the T1 gilts exhibited a greater NDF digestibility than the T6 gilts (*P* < 0.05). The nitrogen balance analysis revealed that the T1 gilts presented decreased urine excretion of nitrogen (− 8.17 g/d, *P* < 0.05) and higher nitrogen retention (+ 9.81 g/d, *P* < 0.05), and thus exhibited higher nitrogen utilization than the T6 gilts. The time-course dynamics of glucose, α-amino nitrogen, urea, lactate, and insulin levels in serum revealed that the T1 group exhibited higher utilization of nutrients after a meal than the T2 or T6 gilts. The T1 gilts also had a higher acetate content and SCFAs in feces than the T6 gilts (*P* < 0.05). The age, body weight and backfat thickness of the gilts at first estrous expression were not affected by the meal frequency, but the gilts in the T1 group had higher levels of serum luteinizing hormone on the 18th day of the 3rd estrus cycle and 17β-estradiol, a larger number of growing follicles and corpora lutea, and higher mRNA expression levels of genes related to follicular development on the 19th day of the 3rd estrus cycle.

**Conclusions:**

The current findings revealed the benefits of a lower meal frequency equal feed intake on nutrient utilization and reproductive function in replacement gilts, and thus provide new insights into the nutritional strategy for replacement gilts, and the dietary pattern for other mammals, such as humans.

## Background

With a replacement rate of 40–50%, the development and management of gilts is considered a crucial factor influencing the reproductive outcome in a sow herd [1]. Successful gilt replacement requires not only sound growth performance but also suitable activation of the hypothalamic-pituitary-gonadal (HPG) axis. The HPG axis, in turn, affects the pulsatile release of reproductive hormones, such as gonadotropin releasing-hormone (GnRH), follicle-stimulating hormone (FSH), luteinizing hormone (LH), and estradiol (E_2_) [1, 2]. Interestingly, activation of the HPG axis only occurs under a nutrition favorable situation, and the metabolic status plays an important role in regulating the secretion of gonadotropins [3–5], which suggests an opportunity to control the HPG axis of gilts through nutritional strategies.

Currently, replacement gilts are typically fed *ad libitum*, however, a growing body of evidence reveals that this free eating pattern is associated with an increased prevalence of metabolic diseases in humans and rodents [6–8], and in contrast, studies of laboratory animals and humans have shown that a decreased meal frequency (MF) can increase insulin sensitivity and thereby reduce obesity related metabolic diseases, and even extend the lifespan [7–9]. Furthermore, a decreased MF changes the diurnal dynamics and even shifts the gut microbiome which has benefits for metabolic health [10, 11]. Decreasing the MF from 12 meals per day to 2 meals per day for 3 or 8 weeks can alter the nutrient utilization, and inflammation status of 30-kg or 60-kg growing male pigs [9, 12]. This evidence indicates that the MF might play a critical role in the female reproductive system because a body of experimental evidence indicates a fundamental link between the metabolic status and the normal estrus cycle [3–5]. In our previous research, time-restricted feeding rescues female mice from body weight gain and glucose intolerance, as well as from the ovarian follicle loss and dysfunction of estrus cyclicity induced by a high-fat diet [13]. However, there is currently no clear evidence showing the effect of MF on the HPG axis in gilts. Therefore, the objective of this study was to investigate the effect of MF on growth preference, short-chain fatty acids (SCFAs) production by the gut microbiota, the post-meal dynamics in the metabolic status, reproductive hormone secretion, and the ovarian follicular development in gilts.

## Materials and methods

All experimental procedures followed the regulations of the Animal Care and Use Committee of Sichuan Agricultural University (S20174302), and were in accordance with the National Research Council’s Guide for the Care and Use of Laboratory Animals.

### Animals and experimental design

Thirty-six Landrace × Yorkshire crossbred gilts with an initially similar body weight (77.6 ± 3.8 kg) and a similar age (150 ± 3 d) were fed the same corn-soybean meal based diet (Table 1) at one of three meal frequencies (*n* = 12) for 14 consecutive weeks: one meal per day (T1); two meals per day (T2), and six meals per day (T6). The daily feed allowances were 2.2 kg from the 1st week to the 10th week to provide 7.44 Mcal DE and 17.6 g SID lysine per day, and 2.4 kg from the 11th week to the end of the experiment (approximately the 14th week) to provide 8.11 Mcal DE and 19.2 g SID lysine per day, which according to the nutrient requirement of swine by the NRC (2012), would result in an average daily growth of approximately 650 g/d, a common practical growth rate. The gilts in the T1 group had access to feed from 08:00 to 11:00; the gilts in the T2 group had access to half of the feed from 08:00 to 09:30 and the other half from 14:00 to 15:30; and the gilts in the T6 group had access to one-six of the feed during the time ranges of 08:00–08:30, 11:00–11:30, 14:00–14:30, 17:00–17:30, 19:00–19:30, and 21:00–21:30. Water was provided *ad libitum*. The gilts were housed individually in each pen, and the pens located in an environmentally controlled room with a temperature in the range of 18 °C to 22 °C.

**Table 1:** Ingredients and nutrient content of experimental diets

Ingredients	kg/t
Corn	708.38
Soybean meal (44%CP)	160.00
Fish meal (65%CP)	30.00
Soybean oil	13.00
Wheat bran	33.40
Corn starch	27.42
*L*-Lysine sulfate, (98.5%)	1.20
*DL*-Methionine, (99.0%)	0.10
Limestone	6.00
Calcium phosphate (dibasic)	11.00
Sodium chloride (feed-grade, 99%)	4.00
Choline chloride (50%)	1.50
Premix^a^	4.00
Total	1000.00
Calculated nutrient levels^b^	
Digestible energy, Mcal/kg	3.38
Crude protein, %	15.76
Calcium, %	0.67
Total phosphorus, %	0.60
STTD phosphorus, %	0.38
SID-Lysine	0.80
SID-(Met+Cys)	0.45
SID-Threonine	0.48
SID-Tryptophan	0.15

^a^Provided per kg of diet: Cu, 20 mg as copper sulfate; Fe, 80 mg as ferrous sulfate; Zn, 100 mg as zinc sulfate; Mn, 25 mg as manganese sulfate; Se, 0.15 mg as sodium selenite; I, 0.14 mg as potassium iodide, vitamin A, 4000 IU; vitamin D_3_, 800 IU; vitamin E, 441 IU; menadione, 0.5 mg; thiamine, 1.0 mg; riboflavin, 3.75 mg; vitamin B_6_, 1.0 mg; vitamin B_12_, 15 μg niacin, 10 mg; *D*-pantothenic acid, 12 mg; folic acid, 1.3 mg; *D*-biotin, 200 μg

^b^Calculated values based on China Feed Information Database 2013

### Measurement of growth traits

The initial body weight of the gilts was measured on the morning of day 1 of the experiment after overnight fasting. The fasted body weight was determined every 2 weeks in the morning before the first meal. The back-fat thickness was measured to equal 65 mm on both sides of the dorsal midline at the last rib using an ultrasound scanner with a Lean Meater (Renco-Lean Meater, Minneapolis, MN, USA). Both side measurements were averaged to obtain a final value.

### Estrous detection

Estrous detection was conducted at the beginning of the experiment and throughout the trial. The gilts were permitted to come into heat naturally to allow measurement of their age at first estrous expression and to evaluate the timing of puberty. As previously characterized [14, 15], all the gilts were exposed (with fence) to mature boars to encourage pubertal estrous. Estrous was detected by only one experienced stockperson based on behavior and vulvar characteristics. The appearance of a pink vulva and vaginal orifice mucus were important signs of estrous initiation, whereas standing still under applied back pressure (standing reflection) was used as an important behavior criterion to establish the onset of first estrous expression. The age, body weight and back-fat thickness at first estrous expression were recorded.

### Nutrient digestibility and nitrogen balance determination

Twenty-four gilts (eight gilts per group) were randomly chosen to conduct an analysis of the nitrogen balance after the gilts received their respective dietary regimens for 10 weeks. The gilts were individually housed in stainless-steel metabolism cages equipped with waterers and feeders and the feeding schedules were kept the same as before. The apparent digestibilities of dry matter (DM), ether extract (EE), crude protein (CP), neutral detergent fiber (NDF), and gross energy, and the nitrogen balance were measured. The gilts were subjected to a 4-day adaptation period and a 5-day sample collection period. During the first and last days of the nitrogen balance trial, 5 g of chromic oxide was added to 100 g of the basal morning meal as a color marker, and the appearance of green color in the faces was used to indicate the start or end of the fecal sample collection period. The freshly collected feces were immediately added with several drops of methylbenzene to prevent fermentation. The fecal samples collected every 24 h were pooled together, weighed, and placed on ice, and 1 mL of HCl (10%) was added to 10 g of feces to prevent nitrogen loss. The urine of each gilt was collected in a plastic container and measured every 24 h from 08:00 on the starting day to 08:00 on the last day of the nitrogen balance trial. Several drops of H_2_SO_4_ (98%) were added to the urine to maintain a pH less than 3.0. and a representative urine subsample (5%) was obtained and stored at 4 °C until analysis. The collected feces and urine samples were pooled together at the end of each nitrogen balance trial. The feces were dried in a force-draft oven (65 °C) for 2 d and then weighed, and the dried feces were ground through a 1-mm screen and frozen until subsequent detection of the DM, EE, energy, NDF and nitrogen content. DM, ether extract (EE), CP, and NDF was determined using the AOAC procedure (2000). The nitrogen content of the diets, urine and feces was determined by the combustion method using a Leco TruSpec analyzer. The gross energy of the feed and feces was determined with a Parr 6400 Bomb Calorimetry (Parr Instrument Co., Moline, IL, USA).

### Fecal sample collection and analysis

After 12 weeks of dietary treatment, fresh feces were collected directly by massaging the rectum of each gilt every 3 h during a 24-h period. The freshly collected samples were then transported in solidified carbon dioxide and stored at − 80 °C until analysis. The SCFAs concentrations in the feces were analyzed through a gas chromatographic method as previously described [13]. Briefly, 0.7 g of fecal sample was suspended in 1.5 mL of distilled water, mixed, and allowed to stand for 30 min. Afterward, the samples were centrifuged at 15,000×*g* and 4 °C for 15 min. One milliliter of supernatant was transferred and mixed with 0.2 mL of metaphosphoric acid (25%, w/v) and 23.3 μL of crotonic acid (210 mmol/L, internal standard). The resulting mixtures were incubated at 4 °C for 30 min, and centrifuged at 15,000×*g* for 10 min. After centrifugation, 0.3 mL of liquid was transferred and mixed with 0.9 mL of chromatographic methanol (1:3 dilution), and the mixture was centrifuged at 10,000×*g* for 5 min. An aliquot of the supernatant (1 μL) was analyzed using a gas chromatography (Varian CP-3800 GC, USA).

### Blood sample collection and analysis

A meal test was conducted after the gilts received their respective dietary regimens for 12 weeks. For the meal test trial, the first blood sample was obtained at 07:50, and 800 g of the corresponding diet was then provided. After complete consumption of the meal (0 min), blood samplings were performed every 10 min during the first hour, every 15 min during the second hour and every 30 min during the third hour. The blood samples were centrifuged at 2500×*g* and 4 °C for 30 min to collect serum, and the serum was stored at − 20 °C for future analysis. The serum glucose, lactate, and urea levels were measured with an automatic biochemical analyzer (7020; Hitachi, Tokyo, Japan) using corresponding commercial analysis kits (Beijing Strong Biotechnologies, Beijing, China) according to the manufacturer’s instructions. The serum α-amino nitrogen concentration was measured using a colorimetric method. The serum insulin levels were measured with commercial ELISA kits (KE10032, Proteintech Group, Rosemont, PA, USA) according to the manufacturer’s instructions.

For determination of the serum FGF21 levels, blood samples were collected at 08:00, 12:00, 16:00 and 20:00 before feeding. The serum FGF21 levels were measured by ELISA according to the manufacturer’s instructions (RD291108200R, BioVendor, LA, USA). Additional blood samples were collected at 30 min intervals for 4 h on the 18th day of the 3rd estrous cycle. The serum LH (B162447) and FSH (B162448) concentrations were measured using commercial ELISA kits, according to the manufacturer’s instructions (BIM, San Francisco, CA, USA). On the 19th day of the 3rd estrous cycle, blood samples were collected to measure the serum 17β-estradiol levels (KGE014, R&D Systems; Bio-Techne, Minneapolis, MN, USA). The blood samples were centrifuged at 2500×*g* and 4 °C for 30 min to collect serum, and the serum samples were stored at − 20 °C for future analysis.

### Ovarian sample collection and analysis

On the 19th day of the 3rd estrous cycle, five gilts from each group were randomly selected for the collection of bilateral ovaries under anesthesia by Shumianning (combination of ketamine, xylazine and midazolam, 2 mL per gilt) from Nanjing Agricultural University. The ovaries were washed three times with ice-cooled phosphate buffered saline (PBS). The cumulus-oocytes complex (COC) was obtained from antral follicles with diameters larger than 3 mm as previously described [16], snap-frozen and stored at − 80 °C. The right ovary was fixed in 4% paraformaldehyde (100 mmol/L phosphate buffer, pH 7.4). The ovaries were fixed with 4% paraformaldehyde in PBS, dehydrated, embedded in paraffin, sectioned (5-μm thickness) and stained with hematoxylin and eosin (HE). The HE-stained sections were examined under microscope (Nikon 80i). The ovarian follicles, including primordial follicles, primary follicles, secondary follicles, and antral follicles, were quantified as previously described [17]. To prevent duplicate counting of follicles, six sections at least 500 μm apart were stained. In the assessment of the primordial, primary, and secondary follicles, only those with visible nuclei were counted, and those without nuclei were not counted. During the counting of antral follicles with diameters less than 1 mm, which are not easy to find in ovarian sections, those with visible oocytes were counted even if they did not have a visible nucleus. The area of the HE-stained sections was estimated with Image-Pro Plus for Windows 6.0 (Media Cybernetics, Maryland, MD, USA). The number of follicles at each stage was normalized by the area of HE-stained ovarian tissue in the sections and is presented as the number of follicles per cm^2^ as previously described [18].

### RNA extraction and gene expression analysis

RNA extraction and real-time PCR were performed as previously reported [19]. Briefly, RNA from each ovary was extracted by TRIzol (15596018; Thermo Fisher Scientific) and purified using RNA mini-columns (RR037A; Takara Bio, Kusatsu, Japan). Reverse transcription and SYBR green quantitative PCR (RR820A; Takara Bio) were performed according to the manufacturer’s protocols. The target primer sequences are shown in Table 2.

**Table 2:** Sequence of primers used for qPCR

Genes	Forward	Reverse
*β-ACTIN*	GGCCGCACCACTGGCATTGTCAT	AGGTCCAGACGCAGGATGGCG
*FSHR*	TCACAGTCCCTCGGTTCCTT	AGCATCACAGCCTGCTCCA
*LHCGR*	ATGGGGCTCTACCTGCTACTCA	GAGCCACCCTCCAAGCATAA
*GDF9*	CTCTGCCTCTTCCTCCTCCACTG	GGTGAATGAGTACGGTGCTCTTGG
*BMP15*	ACCATGCCATCATTCAGAGCCTTG	CGTTGGTCTCAATCAGGAGGATGC
*CYP11A1*	GGCTCCAGAGGCCATAAAGA	ACTCAAAGGCGAAGCGAAAC
*STAR*	GACTTTGTGAGTGTCGGCTGTA	ATCCCTTGAGGTCAATGCTG
*3β-HSD*	CACTGACCTGGGCTGATGAC	GTGGCGAGAAGCAGACAAGA
*CYP17A1*	TCCAAGCCAAGACGAAC	TTTACCACAGAGGCAGAAG

*Abbreviations*: *FSHR* follicle-stimulating hormone receptor, *LHCGR* luteinizing hormone/chorionic gonadotropin receptor, *GDF9* growth differentiation factor 9, *BMP15* bone morphogenetic protein 15, *CYP11A1* cytochrome P450 family 11 subfamily A member 1, *STAR* steroidogenic acute regulatory protein, *3βHSD* 3 β-hydroxysteroid dehydrogenase, *CYP17A1* cytochrome P450 family 17 subfamily A member 1

### Statistical analysis

The experiment design is completely randomized design, and individual pig served as the experimental unit for all analyses. The area under the curve (AUC) was calculated using Prism 6 software (GraphPad Software, La Jolla, CA, USA). The data are presented as the means ± SEMs and were analyzed by one-way analysis of variance (ANOVA) with Tukey’s test for multiple comparisons using Prism 6 software to determine the differences between the groups. The serum nutrient and hormone concentrations obtained from the serial serum sampling were analyzed using the MIXED procedure with the meal frequency, replicates, a repeated statement for the sampling time, and the interaction between meal frequency and time as the main effects (SAS Inst. Inc., Cary, NC, USA). The effect of meal frequency is the fixed effect. The data for the time of first estrous expression were analyzed using Kaplan-Meier statistics. The statistical significance was set at *P* < 0.05.

## Results

### Effects of meal frequency on the growth performance of gilts

In the present study, all the gilts consumed their feed completely during the given feeding period. Thus, all the gilts consumed similar amounts of feed every day. As shown in Table 3, gilts in the T1 group had a higher body weight than the T6 gilts (+ 4.9 kg, *P* < 0.05). The average daily gain of the gilts in the T6 group was greater than that of T1 gilts during weeks 0 to 2 (*P* < 0.05), and the average daily gain of the gilts in the T1 group was greater than that of the T6 gilts during weeks 6 to 8, and weeks 0 to 14 of the experiment (*P* < 0.05). The gilts fed one meal a day showed an increased backfat thickness at the end of week 8 (+ 1.57 mm, *P* < 0.05), compared with those fed six meals a day.

**Table 3:** Effects of meal frequency on growth performance of gilt^1,2,3^

	T1	T2	T6	*P*-value
Body weight, kg				
Initial	77.7 ± 0.92	77.9 ± 1.07	77.6 ± 1.63	0.980
2nd week	86.9 ± 1.19	88.8 ± 1.22	88.3 ± 1.67	0.612
4th week	100.2 ± 1.50	101.1 ± 1.07	100.3 ± 1.73	0.889
6th week	110.1 ± 1.46	111.3 ± 1.28	109.7 ± 1.76	0.735
8th week	120.3 ± 1.35	119.9 ± 1.59	118.0 ± 1.91	0.580
10th week	128.2 ± 1.20	127.1 ± 1.47	124.7 ± 1.65	0.237
12th week	137.5 ± 1.24	136.0 ± 1.74	133.0 ± 1.58	0.132
14th week	146.5 ± 1.43^a^	143.9 ± 1.85^ab^	141.6 ± 1.72^b^	0.042
ADG, g/d				
Week 0–2	763.9 ± 34.44^a^	901.4 ± 28.01^b^	896.5 ± 27.32^b^	0.004
Week 2–4	832.3 ± 26.18	771.9 ± 30.23	744.3 ± 29.75	0.102
Week 4–6	703.6 ± 39.87	728.6 ± 32.94	673.8 ± 22.37	0.499
Week 6–8	727.4 ± 42.80^a^	611.9 ± 35.34^b^	592.9 ± 31.77^b^	0.031
Week 8–10	567.9 ± 28.73	517.9 ± 28.58	481.0 ± 29.96	0.122
Week 10–12	662.5 ± 37.88	631.5 ± 40.34	594.6 ± 31.64	0.436
Week 12–14	642.9 ± 78.89	569.6 ± 64.97	612.5 ± 29.22	0.700
Week 0–14	701.5 ± 8.73^a^	673.5 ± 13.03^ab^	653.4 ± 8.74^b^	0.009
Backfat thickness, mm				
Initial	10.01 ± 0.41	9.98 ± 0.31	9.94 ± 0.51	0.962
2nd week	10.45 ± 0.21	10.55 ± 0.35	10.73 ± 0.46	0.846
4th week	11.73 ± 0.28	11.41 ± 0.29	11.37 ± 0.50	0.758
6th week	12.70 ± 0.32	11.70 ± 0.60	11.89 ± 0.59	0.356
8th week	13.94 ± 0.49^a^	12.08 ± 0.48^ab^	12.37 ± 0.58^b^	0.035
10th week	14.21 ± 0.44	12.83 ± 0.64	12.60 ± 0.64	0.113
12th week	15.26 ± 0.56	14.44 ± 0.74	13.56 ± 0.64	0.169
14th week	16.36 ± 0.36	15.72 ± 0.67	15.08 ± 0.65	0.215

^1^Results are presented as means ± SEMs. *n* = 12

^2^T1 means gilts fed one meal per day, T2 means gilts fed two meals per day, and T6 means gilts fed six meals per day

^3^Different letters ^a, b^ denote *P <* 0.05

### Effects of meal frequency on the nutrient digestibility and the nitrogen balance

The effects of MF on the apparent total tract digestibility of energy, EE, CP, and NDF of each gilt are presented in Table 4. The MF had no effects on the digestibility of DM, CP, EE, ash, or gross energy. Interestingly, the digestibility of NDF obtained for the T1 pigs was higher than that found for the T6 pigs. The NDF digestibility of the T2 gilts was between those of the T1 and T6 gilts and was not affected by MF. As shown in Table 5, the MF had no effect on the nitrogen digestibility and fecal nitrogen excretion, but the urine excretion obtained from the T6 gilts was higher than that obtained with the T1 gilts. The T1 gilts had an increased amount of retained nitrogen and presented a higher biological value of nitrogen.

**Table 4:** Effects of meal frequency on the apparent total tract digestibility of nutrients in gilts^1,2,3^

Items	T1	T2	T6	*P*-value
DM, %	90.8 ± 0.013	89.8 ± 0.003	90.0 ± 0.004	0.953
CP, %	91.0 ± 0.013	89.0 ± 0.013	87.9 ± 0.023	0.879
EE, %	80.5 ± 0.027	80.3 ± 0.016	77.0 ± 0.020	0.478
Ash, %	42.7 ± 0.048	47.6 ± 0.019	46.6 ± 0.020	0.529
NDF, %	65.2 ± 0.038^a^	57.7 ± 0.022^ab^	54.4 ± 0.021^b^	0.042
Gross energy, %	92.6 ± 0.008	90.8 ± 0.005	90.7 ± 0.003	0.065

^1^Results are presented as means ± SEMs. *n* = 8

^2^T1 means gilts fed one meal per day, T2 means gilts fed two meals per day, and T6 means gilts fed six meals per day

^3^Different letters ^a, b^ denote *P <* 0.05

**Table 5:** Effects of meal frequency on the nitrogen balance in gilts^1,2,3^

Items	T1	T2	T6	*P*-value
Nitrogen balance, g/d				
Intake	70.04	70.04	70.04	1.00
Fecal excretion	6.29 ± 0.93	7.68 ± 0.88	8.50 ± 1.59	0.408
Urine excretion	24.09 ± 0.99^a^	32.02 ± 2.14^b^	32.26 ± 3.32^b^	0.027
Absorbed N	63.74 ± 0.93	62.36 ± 0.88	61.54 ± 1.59	0.408
Retained N	39.08 ± 1.50^a^	30.34 ± 2.55^b^	29.27 ± 3.94^b^	0.040
N net utilization, %^4^	55.80 ± 2.14^a^	43.32 ± 3.64^b^	41.80 ± 5.63^b^	0.039
N digestibility, %	91.01 ± 1.33	89.04 ± 1.26	91.75 ± 1.31	0.189
Retention ratio, %^5^	61.31 ± 2.16^a^	48.46 ± 3.80^b^	47.20 ± 5.83^b^	0.044

^1^Results are presented as means ± SEMs. *n* = 8

^2^T1 means gilts fed one meal per day, T2 means gilts fed two meals per day, and T6 means gilts fed six meals per day

^3^Different letters ^a, b^ denote *P <* 0.05

^4^N net utilization, % = N retained/N intake × 100

^5^Retention ratio, % = N retained/N absorbed × 100

### Effects of meal frequency on gut metabolite production by the gut microbiota in gilts

**Fig. 1:**
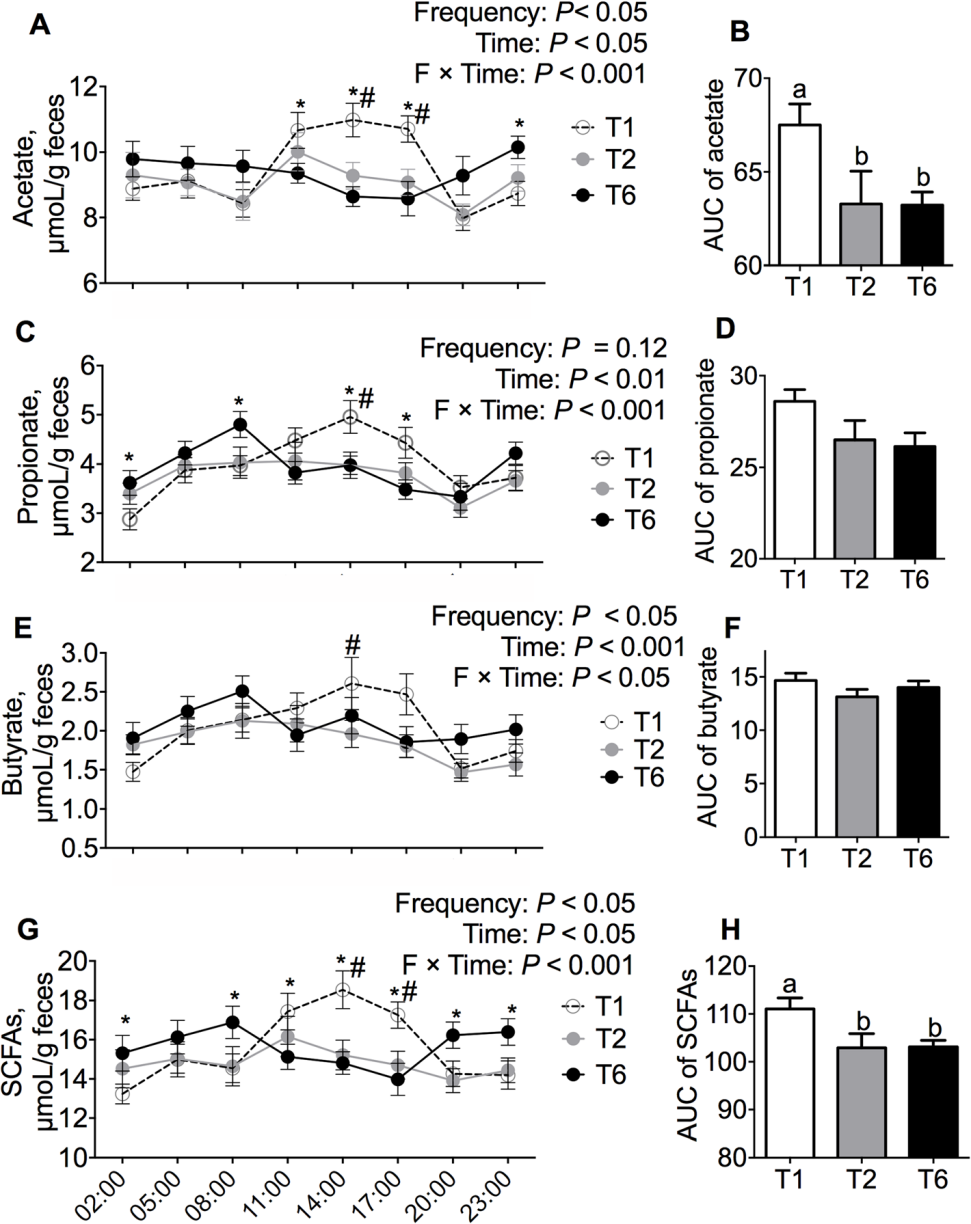
Effects of meal frequency on the production of microbial metabolites in gilts. After 12 weeks of treatment, fresh feces were collected from each gilt at 3-h intervals for a 1-day period. The fecal acetate (**a**), propionate (**c**), butyrate (**e**) and total SCFAs (**g**) production and the area under the curve (AUC) of acetate (**b**), propionate (**d**), butyrate (**f**) and total SCFAs (**h**) are shown. (*n* = 12). Labeled means without a common letter differ, *P* < 0.05. *P < 0.05 (T1 vs. T6); ^#^*P* < 0.05 (T1 vs. T2)

Feces were collected every 3 h to detect the concentrations of the acetate, propionate, and butyrate (Fig. 1a-h). Interestingly, we observed time-course changes in the SCFAs concentrations in feces at different time points (*P* < 0.05, Fig. 1a, c, e and g). The acetate contents in the feces of the T1 gilts sampled at 11:00, 14:00 and 17:00 were higher than those found in the corresponding samples collected from the gilts in the T2 and T6 groups (*P* < 0.05, Fig. 1a), but the acetate content in the feces of the T6 gilts collected at 23:00 was higher than that in the corresponding fecal samples from the T1 gilts (*P* < 0.05, Fig. 1a). The AUC of the acetate contents in feces from the T1 gilts was higher than those found in the other two groups (*P* < 0.05, Fig. 1b). The propionate contents in feces at different time points were differentially affected by MF (Fig. 1c). The fecal propionate contents at 02:00, 08:00, 14:00 and 17:00 showed difference between the T1 and T6 groups (*P* < 0.05, Fig. 1c). The fecal propionate contents obtained from the T1 and T2 groups at 14:00 were different (*P* < 0.05, Fig. 1c). The AUC of the propionate contents in feces not affected by the MF (*P* > 0.05, Fig. 1d). The butyrate contents in the fecal sample collected at 14:00 showed difference between the T1 and T2 groups (*P* < 0.05, Fig. 1e). The AUC of the butyrate contents in feces was not affected by the MF (*P* > 0.05, Fig. 1f). The content of total SCFAs, sum of the acetate, propionate, and butyrate contents, in the fecal samples collected at 02:00, 08:00, 11:00, 14:00, 17:00, 20:00, and 23:00 were differentially affected by the MF (*P* < 0.05, Fig. 1g). The total SCFAs contents in the fecal samples obtained from the T1 gilts presented a higher AUC than those obtained for the other two groups (*P* < 0.05, Fig. 1h).

### Effects of meal frequency on blood metabolites in gilts

**Fig. 2:**
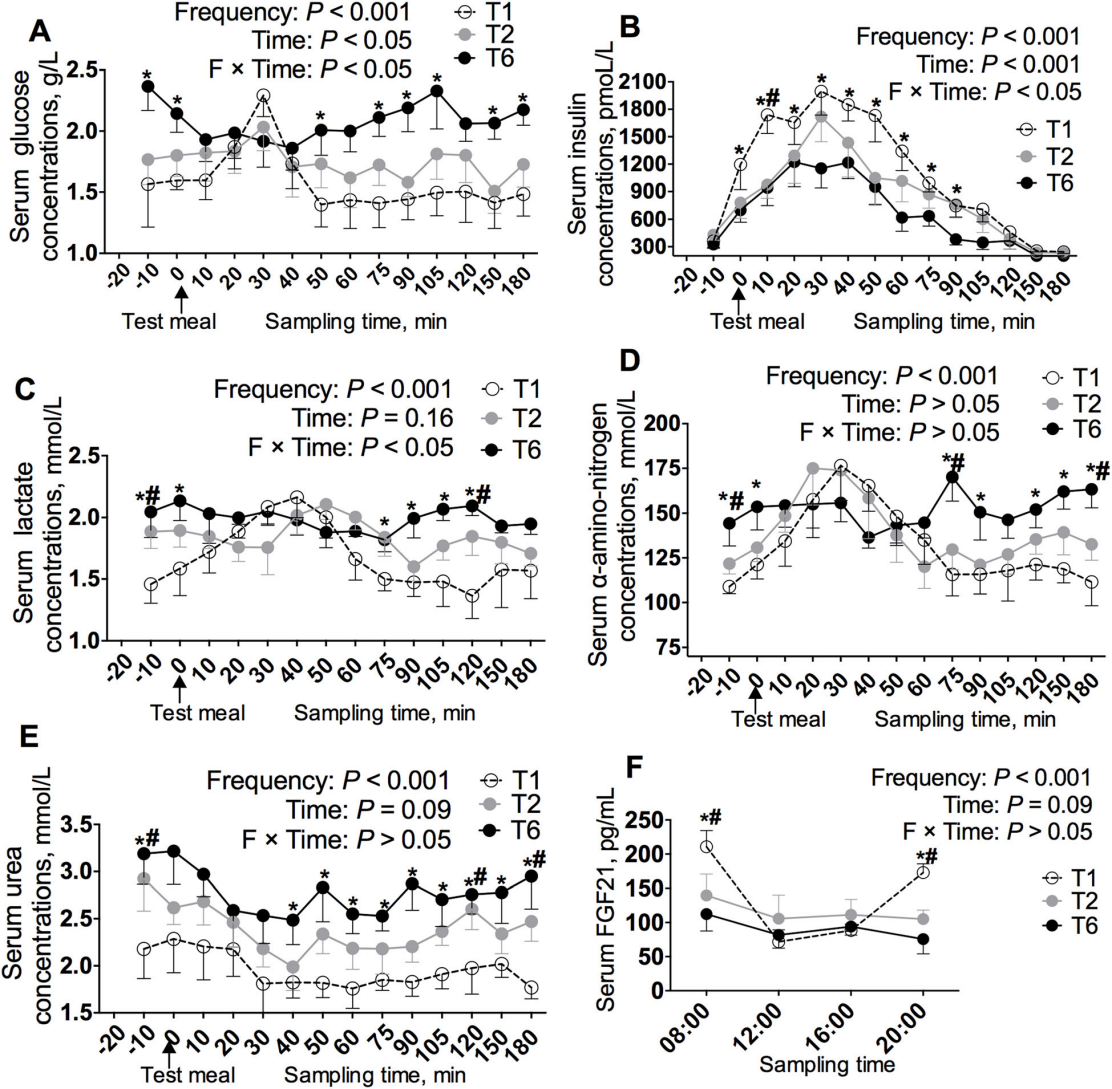
Effect of meal frequency on the metabolic status after a test meal. Postprandial changes in the serum glucose (**a**), insulin (**b**), lactate (**c**), α-amino-nitrogen (**d**) and urea (**e**) concentrations after a test meal during the 12 preceding weeks (*n* = 5–6). (**f**) Serum FGF21 levels after respective dietary regimens for 12 weeks (*n* = 8). *P < 0.05 (T1 vs. T6); ^#^ *P* < 0.05 (T1 vs. T2)

To determine whether the MF altered the metabolic state and feed utilization over the course of the postprandial phase, the serum concentrations of glucose, insulin, lactate, α-amino nitrogen, and urea before and after the morning meal were detected, and the results are presented in Fig. 2a-e. The gilts in the T6 group had higher serum glucose levels than in the T1 gilts at the time points of − 10, 0, 50, 75, 90, 105, 150, and 180 min in the meal test (*P* < 0.05, Fig. 2a). In the T1 gilts, the serum glucose concentration increased 10 min after meal ingestion until reaching a peak 30 min postprandially, and then decreased to values similar to the baseline, which is quite different from the results found in the T6 gilts (Fig. 2a). The MF also changed the postprandial serum insulin concentrations until reaching a peak all the three groups reached a peak 30 min after the test meal, and 0, 10, 20, 30, 40, 50, 60, 75, and 90 min after the test meal, the T1 gilts had higher serum insulin concentrations than those in the T6 group (*P* < 0.05, Fig. 2b). The serum lactate concentrations after a test meal were affected by the MF in the T1 gilts (time effects: *P* < 0.05, Fig. 2c) but not in the T6 gilts. The serum lactate concentrations at − 10, 0, 75, 90, 105, and 120 min after a test meal were differentially affected by the MF (*P* < 0.05, Fig. 2c). In the T1 and T2 gilts, the serum α-amino nitrogen reached a peak at 30 min postprandially (time effects: *P* < 0.05, Fig. 2d). However, in the T6 gilts, the serum α-amino nitrogen levels were not affected by meal ingestion (time effects: *P* > 0.05, Fig. 2d). With the exception of the peak, the serum concentrations of α-amino nitrogen were differentially altered by the MF at − 10, 0, 75, 90, 120, 150, and 180 min after a test meal (*P* < 0.05, Fig. 2d). The serum urea concentration did not vary over time after meal ingestion (time effects: *P* = 0.09, Fig. 2e). The urea levels in the T1 gilts were lower than those in the T6 gilts (*P* < 0.05, Fig. 2e). The gilts in the T1 group had higher serum FGF21 levels than in the T2 and T6 group gilts at the time points of 08:00 and 20:00 (*P* < 0.05, Fig. 2f).

### Effects of meal frequency on the secretion of luteinizing hormone and ovarian follicular development

**Fig. 3:**
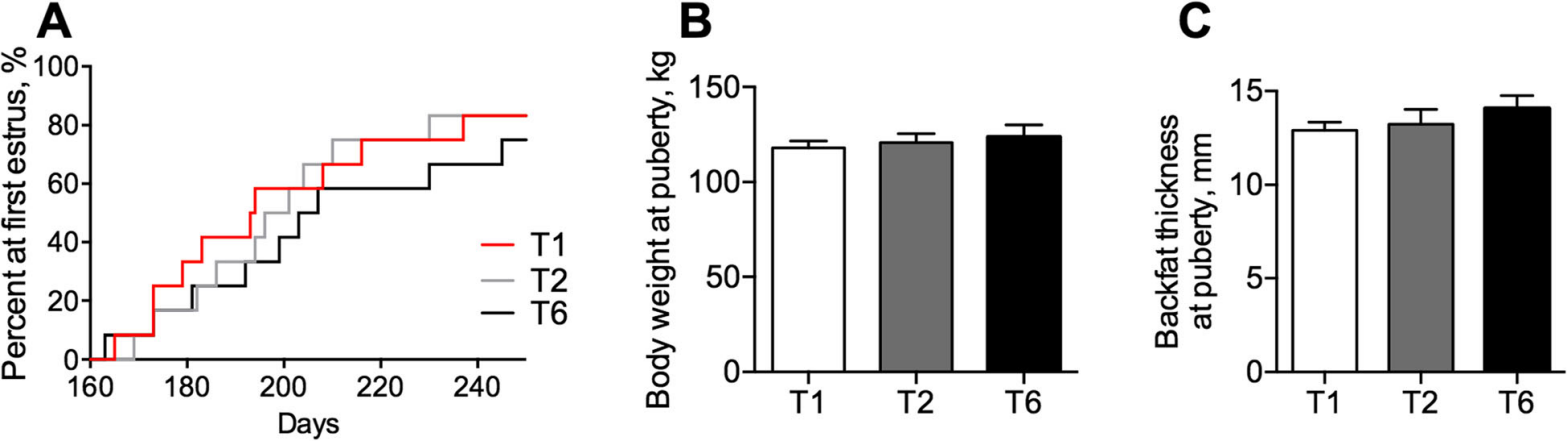
Effects of meal frequency on first estrous expression performance. The age (**a**), body weight (**b**), and backfat thickness (**c**) on the day at which the gilts reached the first estrous expression (*n* = 12)

**Fig. 4:**
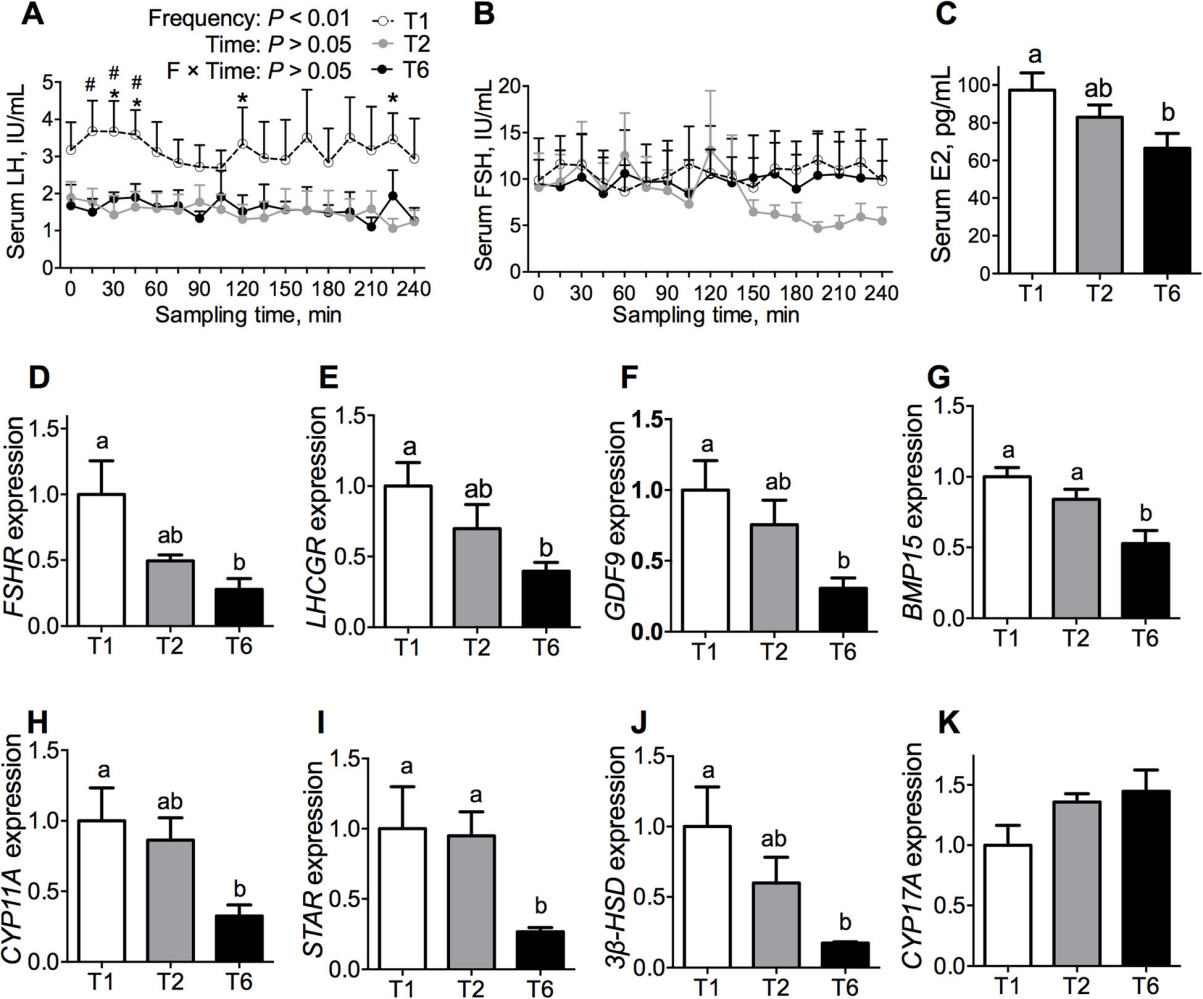
Effects of meal frequency on follicular development in gilts. Serum LH (**a**) and FSH (**b**) levels on the 18th day of the 3rd estrous cycle (*n* = 6–8). **c** Serum 17β-estradiol levels (*n* = 6–8) and relative gene expression levels of *FSHR* (**d**), *LHCGR* (**e**), *GDF9* (**f**), *BMP15* (**g**), *CYP11A* (**h**), *STAR* (**i**), *3 β-HSD* (**j**) and *CYP17A* (**k**) in the cumulus-oocytes complex on the 19th day of the 3rd estrous cycle (*n* = 5). Labeled means without a common letter differ, *P* < 0.05. *P < 0.05 (T1 vs. T6); ^#^ *P* < 0.05 (T1 vs. T2)

The age of first estrous expression, body weight and backfat thickness at first estrous expression were not affected by the MF (*P* > 0.05, Fig. 3a-c). The rates of the T1 and T6 gilts that reached first estrous expression by an age of 180, 200, and 250 days were 41.67% and 8.33%, 66.67% and 41.67%, and 91.67% and 75%, respectively, but the differences were not significant (*P* > 0.05, Fig. 3a). To further evaluate whether the MF has an effect on ovarian follicular development, we investigated the serum reproductive hormone levels on the 18th day of the third estrous cycle. The gilts in the T1 group exhibited substantially higher LH concentrations than those in the T2 and T6 groups at different time points on the 18th day of the 3rd estrus cycle (*P* < 0.05, Fig. 4a), but no marked differences in the FSH levels were detected (*P* > 0.05, Fig. 4b). On the 19th day of the 3rd estrus cycle, the E_2_ concentrations in the T1 gilts were higher than those in the T6 gilts (*P* < 0.05, Fig. 4c).

The expressions of the COC gene were presented in Fig. 4d-k. The mRNA levels of the ovarian gonadotropin receptors *FSHR* and *LHCGR,* were higher in the T1 and T2 gilts than in the T6 gilts (*P* < 0.05, Fig. 4d and e). We then evaluated the expression levels of gene related to ovarian follicular development, including bone morphogenetic protein 15 (*BMP15*) and growth differentiation factor 9 (*GDF9*). The mRNA expression levels of *BMP15* and *GDF9* in gilts was elevated by a lower MF in gilts (Fig. 4f and g). The expression levels of steroidogenic enzymes (*CYP11A1*, cytochrome P450, family 11, subfamily A polypeptide 1; *STAR*, steroidogenic acute regulatory protein and *3β-HSD*, 3-beta (β)-hydroxysteroid dehydrogenase) were increased by a lower MF (*P* < 0.05, Fig. 4h-j). No differences in the expressions of *CYP17A* was affected by the MF (Fig. 4k).

The effects of the MF on the development of follicles in gilts at 3rd estrus are presented in Table 6. The numbers of primordial follicles and antral follicles with diameters between 1 and 3 mm and the numbers of antral follicles with diameters ≥3 mm were not affected by the MF (*P* > 0.05). The number of growing follicles and the sum of the numbers of primary, secondary, and antral follicles (with diameters less 1 mm) were greater in the pigs fed one meal per day than in the gilts fed six meals per day (*P* < 0.05). The comparison of the T1 and T6 gilts revealed that a lower MF increased the number of corpora lutea (*P* < 0.05, Table 6).

**Table 6:** Effects of meal frequency on the development of follicles of gilts at 3rd estrous^1,2,3^

Items	T1	T2	T6	*P*-value
No. of follicle^4^ per cm^2^				
Primordial follicle	11.80 ± 1.02	10.97 ± 0.75	10.77 ± 1.08	0.732
Growing follicle	12.37 ± 0.78^a^	10.37 ± 1.04^ab^	8.13 ± 0.79^b^	0.016
Total follicles	24.17 ± 1.38	21.64 ± 1.39	18.91 ± 1.49	0.087
No. of visible follicle^5^, *n*				
Diameter 1 ~ 3 mm	29.20 ± 1.69	27.60 ± 1.08	28.40 ± 1.81	0.772
Diameter ≥ 3 mm	32.80 ± 3.88	30.00 ± 3.74	29 ± 1.82	0.705
No. of corpora lutea, *n*	28.80 ± 3.22^a^	22.60 ± 1.12^ab^	18.20 ± 0.66^b^	0.048

^1^Results are presented as means ± SEMs. *n* = 5

^2^T1 means gilts fed one meal per day, T2 means gilts fed two meals per day, and T6 means gilts fed six meals per day

^3^ Different letters ^a, b^ denote *P <* 0.05

^4^Growing follicle, sum of numbers of primary follicle, secondary follicle, and antral follicle (with diameter below 1 mm) detected by HE staining

^5^Number of follicles detected by visible measurement

## Discussion

The reproductive fitness of gilts is tightly linked to the metabolic status, and an imbalance in metabolism can cause reproductive disorders and even infertility [3, 20]. The successful cultivation of replacement gilts requires not only sound growth performance, but also suitable activation of the HPG axis to activate pubertal onset and maintain the normal estrous cycle [21]. The meal frequency played a critical role in metabolic status in pigs, humans, and rodents [6–8]. Less MF can affect growth, and alleviate high-fat diet-induced lipid accumulation and inflammation in adipose tissue in pigs [12, 22]. In this study, we tested the hypothesis that a lower MF could influence both the nutrient utilization and reproductive processes of replacement gilts, and the current findings revealed that a lower MF increased the nutrient utilization, promoted the secretion of luteinizing hormones, and improved the ovarian follicular development in gilts. To the best of our knowledge, the results of this study offer a linkage between dietary patterns and the development of peripheral and reproductive tissues, and will thus provide new insights into nutritional strategies for gilt replacement.

Currently, replacement gilts are typically fed *ad libitum*, and the gilts might visit the feeders up to 15 times per day [23]. This eating pattern encourages gilts to extend their daily nutrient intake period and thereby shorten the fasting period [1]. However, a growing body of evidence reveals that this free eating pattern is associated with negative effects on growth, adipose tissues inflammation and systemic health in growing pigs [12, 22]. In this study, gilts were fed one, two, or six meals per day, but the three dietary treatment groups were given same eating windows of 3 h, which eliminated the differences in energy expenditure (such as heat increment) induced by variations in the feeding duration. Preliminary results revealed that gestating gilts or sows fed two or six meals per day during gestation show different on reproductive outcomes [24], and the available evidence also reveals that growing pigs fed one meals, or two meals per day, or with free access to feed presented different nutrient digestibilities and differential adipose tissue metabolism [25–27]. Based on the above-mentioned studies, the MF were set to one, two or six meals per day in the present study. In agreement with previous studies [12], pigs fed at a lower MF presented an increased ADG and a higher final body weight (*P* < 0.05, Table 3). In this study, the feed intake was similar among the three groups, and the increased body weight were observed in the T1 gilts suggested that a lower MF increased the nutrient utilization. In the present study, the T1 gilts showed faster glucose clearance with an increased serum insulin concentration (*P* < 0.05, Fig. 2a and b), which agrees with the results of studies in mice and humans [6, 8], and these findings show that limited access to a single meal results in faster removal of glucose from the circulation compared with that found with *ad libitum* access. Glucose is the most important fuel for metabolism, and faster glucose clearance suggests higher glucose utilization. Insulin augments glucose transport in skeletal muscle, adipose tissue, and other tissues, and this finding is also supported by an enhanced body weight gain in the gilts fed at a lower MF. In humans and rodents, a limited MF has beneficial effects on energy homeostasis, which increases energy utilization and decreases lipid accumulation [10, 28]. To our surprise, this study revealed that a lower MF had no effects on the backfat thickness in gilts (*P* > 0.05, Table 3), which suggested that a lower MF did not affect fat deposition in gilts. The main reason for this inconsistency might be attributed to the difference in the age of the animals. The models used in both human and mouse studies are adults, which easily exhibit higher fat deposition [6–8], whereas the gilts used in this study were growing and had not reached sexual maturity and thus exhibit higher lean tissue accretion than adipose tissue [29, 30]. In this study, the gilts fed one meal per day experienced an increased body weight gain, but this MF had no effect on the backfat thickness, which suggests that MF influences lean deposition.

To test whether a lower MF can influence lean mass metabolism, we tested the nitrogen balance in gilts. Indeed, the T1 gilts showed decreased urine excretion (T1 vs. T6: 24.1 g/d vs. 32.2 g/d), and increased amounts of retained nitrogen (T1 vs. T6: 39.1 g/d vs. 29.3 g/d) and higher biological values of nitrogen (T1 vs. T6: 61.3% vs. 47.2%). In addition, the gilts fed one meal per day showed lower serum α-amino nitrogen and urea levels over time after a meal, which suggests that a lower MF improves nitrogen utilization, and the increased body weight gain was likely attributed to lean mass accretion. Indeed, in neonatal pig, intermittent bolus feeding enhances muscle protein synthesis to a greater extent than continuous feeding via the mTOR pathway [31]. Moreover, the MF had no effects on the digestibility of DM, CP, gross energy, EE, and ash were observed, but the NDF digestibility of the gilts fed one meal per day was higher than that of gilts fed six meals per day, which suggests that a low MF increases fiber fermentation (Table 4). The gut microbiota has been recognized as an important factor in regulating host metabolism through SCFAs [10, 32, 33], which stimulated us to test the effect of MF on SCFAs production in feces. Our study showed that the time-course changes of the SCFAs concentrations in feces at different time points showed difference among the various treatment groups (*P* < 0.05, Fig. 1g). In agreement with this change, the interval between meals results in a shift in the gut microbiota composition [10, 11]. The gilts fed one meal per day but not the gilts fed six meals per day showed a cyclic fluctuation in the fecal SCFAs concentration (*P* < 0.05, Fig. 1g). In this study, the T1 feeding regimen increased the acetate and total SCFAs production (*P* < 0.05, Fig. 1a and g). Acetate is utilized for lipogenesis in the liver and as a fuel source once it enters the peripheral circulation [34] and increased SCFAs production promotes energy availability for both the microbiota and host intestine [35, 36], which might contribute to the health of the gastrointestinal tract and peripheral tissues of the host. Taken together, the results showed that one meal per day increases the nutrient utilization and growth performance of gilts.

Pubertal onset is the marker of sexual maturation in replacement gilts, and can be affected by the growth performance of gilts. In the present study, no marked delay in the first observed estrous was found among the three groups (*P* > 0.05, Fig. 3a), and this finding can be attributed to two reasons. First, the dietary treatment began at the age of 150 d and a body weight at 77 kg, which is too late for the gilts to be affected by the dietary treatment. Second, although the gilts fed one meal per day experienced a higher body weight gain and had a higher final body weight than the gilts fed six meals per day at the 14th week of the experiment, the ADG of the gilts fed six meals per day already exceeded the minimum growth rate (600 g/d) required for pubertal maturation. The exposure of females to sexually mature male pheromones leads to the initiation of proestrus, including increased hypothalamic secretion of GnRH and a preovulatory LH surge [2, 37], thus the gonadotropin concentrations were measured on the 18th day of the 3rd estrus cycle. In the present study, we found that the gilts fed one meal per day exhibited a substantially higher LH concentrations than the gilts in the other two groups at different time points (*P* < 0.05, Fig. 4a), but no marked differences in the FSH levels of the gilts were found (*P* > 0.05, Fig. 4b), which suggests that a lower MF promotes the GnRH secretion. Higher concentrations of E_2_ were found in the circulation of the gilts fed one meal per day (Fig. 4c), and this finding was associated with genes related to E_2_ biosynthesis, such as *CYP11A1, STAR,* and *3βHSD* (*P* < 0.05, Fig. 4h-j). The gene expression levels of *GDF9* and *BMP15*, which are indicators of the oocyte quality [38, 39], were also higher expression levels in the gilts fed one meal per day and lower in the gilts fed six meals per day (*P* < 0.05, Fig. 4f and g). The release of reproductive hormones is closely associated with ovarian follicular development. In agreement with the increases in the LH and E_2_ concentrations, the gilts fed one meal per day had a greater number of corpora lutea, an indicator of recently ovulated matured follicles, than the gilts fed six meals per day (*P* < 0.05, Table 6). Interestingly, the gilts fed one meal per day also had a greater number of growing follicles, which suggests that these gilts might have a greater ovarian reserve. Most studies conducted in humans, mice, and pigs revealed that a larger ovarian pool would result in greater reproductive potential and a longer reproductive span [40, 41].

Caloric restriction improves the reproductive preference mainly due to a decrease body weight and attenuation of fat over-deposition [42–44]. In this study, a decreased MF increased the body weight gain without altering the fat deposition (indicated by the backfat thickness). In this study, all the gilts were provided similar nutrient quantities, which is different from the regimen of caloric restriction, the gilts fed at different MF had different periods of fasting and the gilts fed one meal per day had a longer fasting period than those in the other two groups. In contrast, replacement gilts are typically fed *ad libitum*, and this feeding pattern encourages the gilts to extend their daily nutrient intake period and thereby shorten their fasting period. FGF21 was first discovered as a fast-response hormone [45, 46], and was later observed to be induced by a variety of nutritional situations such as a low-protein diet [17, 47, 48], and a ketogenic diet [49, 50], but not by caloric restriction [48]. In our previous research, we found that time-restricted feeding rescues female mice from body weight gain and glucose intolerance as well as from ovarian follicle loss and dysfunction of estrus cyclicity induced by a high-fat diet via liver FGF21 [13]. The different effects of MF and caloric restriction on FGF21 secretion, suggests that the beneficial effects of a lower MF are completely different from caloric restriction. Because the reproductive process is controlled by multilevel regulators [21, 37], the metabolic status is known to be an important factor influencing the function of the HPG axis [3, 20, 51, 52]. It will be interesting to examine which metabolic signals could mediate the effects of meal frequency on the reproductive function of gilts.

## Conclusion

This study, provides evidence showing that MF exerts profound effects on the nutrient utilization and reproductive function, and gilts fed one meal per day showed improved the nutrient utilization and better ovarian follicular development. The results of the present study will provide new insights into nutritional strategies for replacement gilts and into the dietary patterns of other mammals, such as humans.

## Data Availability

All data generated or analyzed during this study are available from the corresponding author on reasonable request.

## Abbreviations

MF: Meal frequency
ADG: Average daily gain
GnRH: Gonadotropin releasing-hormone
FSH: Follicle-stimulating hormone
LH: Luteinizing hormone
SCFAs: Short-chain fatty acids
AUC: Area under a curve
DM: Dry matter
EE: Ether extract
CP: Crude protein
NDF: Neutral detergent fiber
COC: Cumulus-oocytes complex
FSHR: Follicle-stimulating hormone receptor
LHCGR: Luteinizing hormone/chorionic gonadotropin receptor
GDF9: Growth differentiation factor 9
BMP15: Bone morphogenetic protein 15
*CYP11A1*: Cytochrome P450 family 11 subfamily A member 1
STAR: Steroidogenic acute regulatory protein
3β-HSD: 3β-Hydroxysteroid dehydrogenase
*CYP17A1*: Cytochrome P450 family 17 subfamily A member 1
